# Data Quality Implications in Research when Transitioning to a New Electronic Medical Record

**DOI:** 10.23889/ijpds.v9i5.2758

**Published:** 2024-09-18

**Authors:** Zoe Hsu, Cassandra Chisholm, Conné Lategan, Eddy Lang, Xiaoming Wang, Erik Youngson

**Affiliations:** 1 The Alberta Strategy for Patient Oriented Research Support Unit; 2 Provincial Research Data Services, Alberta Health Services; 3 Faculty of Medicine, University of Alberta; 4 Department of Emergency Medicine, University of Calgary

The province of Alberta has been implementing a new hospital based electronic medical record (EMR) over the past several years based on the Epic system. With this implementation comes challenges related to consistent mapping of data to ensure consistency of data over time before and after implementation, even for standardized and routinely used elements.

In a research study evaluating potential disparities in access to Emergency Department (ED) care, particularly in wait times and patient boarding (the time spent in the ED awaiting transfer to an inpatient bed), between mental health (MH) and non-MH patients during the COVID-19 pandemic. An interrupted time series analysis was used to evaluate the impact of the pandemic on boarding time. While there initially appeared to be a large decrease in boarding time near the start of the pandemic, it was later determined to be a data quality issue corresponding to the rollout of the new EMR at one hospital, despite the data coming from a secondary standardized dataset that is used for provincial and national reporting and expected to be reliable.

This presentation will highlight how the issue was identified, what steps were taken to confirm the underlying cause, and how it was corrected to ensure accurate results in this research study.

